# 914. Efficacy of Mental Health Screening in a Multidisciplinary Burn Clinic

**DOI:** 10.1093/jbcr/irag033.439

**Published:** 2026-04-06

**Authors:** Rebecca Altschuler, Erica Whetten, Joya Neal, Snehal Jain, Kevin N Foster

**Affiliations:** Diane & Bruce Halle Arizona Burn Center - Valleywise Health, Phoenix, AZ; Valleywise Health Medical Center, Phoenix, AZ; Diane & Bruce Halle Arizona Burn Center - Valleywise Health, Phoenix, AZ; Creighton University School of Medicine, Phoenix, AZ; Diane & Bruce Halle Arizona Burn Center - Valleywise Health, Phoenix, AZ

## Abstract

**Introduction:**

Burn injuries can lead to mental health sequalae. Early screening is essential to identify psychological needs. Burn clinics use screeners to detect risk for these conditions. The PTSD Checklist for DSM-5 (PCL-5) is validated across trauma populations but is lengthy for high-volume clinics. The Primary Care PTSD Screen (PC-PTSD) offers a shorter alternative, with strong accuracy in medical populations. There is no validated screening measure in burn populations. This study compared the PCL-5 with the PC-PTSD and added depression screening.

**Methods:**

Retrospective data was reviewed from a three-month period. Chi-square analyses compared associations across three levels: accurate, false positive, or symptom sensitivity. The accurate level includes elevated screeners that resulted in PTSD diagnoses. False positive included elevations that did not result in the diagnosis of any mental health disorder. The sensitive level consists of screener scores that are elevated but resulted in diagnoses other than PTSD.

**Results:**

Both groups were significantly associated with diagnoses (*p*<.0001). Residuals were examined to identify which sublevels contribute most to significance. The symptom sensitivity group contributed most to significance (7.97), followed by accuracy (7.22). In the PC-PTSD/PHQ4 sample, the sensitivity level was approximately 53.33%, the accurate level was approximately 33.33% and the false positive level was 13.33%. In the PCL-5 group the sensitivity level was 53.13%, the accurate level was 34.38% and the false positive level was approximately 12.5%.

**Conclusions:**

The PCL-5 and PC-PTSD/PHQ-4 showed similar sensitivity to diagnosis, with limitations including small, uneven samples and clinic workflow changes. Examination of residuals indicated that in the burn clinic setting, both screening tools are more sensitive to symptoms versus accurate diagnoses.

**Applicability of Research to Practice:**

Both screeners detected symptoms more than specific diagnoses. As such, it is imperative for screener tools to be followed by clinical evaluation by a psychologist. Given clinic demands, the 4-item PC-PTSD is more feasible than the 20-item PCL-5. Pairing it with the PHQ-4 expands coverage to depression, increasing utility in fast-paced settings. Further research should refine screening tools in burn care, prioritizing efficiency, accessibility, and clinical relevance.

**Funding for the study:**

N/A.

